# Proust and involuntary retrieval

**DOI:** 10.3389/fpsyg.2024.1235098

**Published:** 2024-02-13

**Authors:** Pascale Gisquet-Verrier, David C. Riccio

**Affiliations:** ^1^NeuroPSI - Institut des Neurosciences Paris-Saclay, Saclay, France; ^2^Department of Psychological Sciences, Kent State University, Kent, OH, United States

**Keywords:** memory, retrieval, reminder, human, animal, Proust (Marcel), voluntary memories, involuntary autobiographic memory

## Abstract

Proust was undoubtedly a pioneer in exploring cognitive processes engaged in memory. The analysis of the episode of the madeleine, as well as the study of Proust’s statements on the goals of his work, clearly reveal the visionary side of this author. Long before several concepts entered into mainstream scientific thought, Proust proposed, among other things, that recall was a reconstruction, that a sensory cue could provoke a memory recall, and that we should distinguish between voluntary and involuntary memory. Through numerous episodes of “*involuntary reminiscence*” scattered throughout his work, Proust illustrates a particular form of autobiographic memory recall: a recall that does not involve consciousness and whose starting point is an emotion provoked by a specific cue. This recall, which leads, according to Proust, to a more intense revival of the memory than voluntary recall, has only reached prominence in cognitive science more than 80 years later. Additionaly, Proust underlined the determinant role that emotion may have in this particular form of recall. On the other hand, studies on animals have shown that the presentation of a retrieval cue could induce emotional reactions followed by a facilitation of the memory retrieval associated with the cue. The existence of these data, which support Proust’s proposals, should encourage the neuroscience community to further explore, in humans and animals, this form of cue elicited emotion that initiated involuntary recall of autobiographical memory.

## Introduction

On the occasion of the 100th anniversary of the death of Marcel Proust, the literary interest of his work has been widely recalled. In *Search of Lost Time* is not only a monumental work, but also a work on memory and time that makes its author one of the first explorers of memory processes, a point already emphasized by Lehrer (2007). In this selective and limited review of the literature, we try to highlight the contribution to memory that was made many years ago by Proust.

It is surprising to note the visionary side of Proust, who proposed concepts that have often been confirmed years later. Revisiting the episode of the madeleine, it becomes clear that Proust described an involuntary retrieval of autobiographical memories which only became systematically investigated some 80 years later by cognitive psychologists. An unconscious process that begins with the encounter of a cue arousing strong emotion and that Proust called “*involuntary reminiscence*.” Since then, the literature offers few examples similar to this type of retrieval in humans, while a close parallel can be drawn between Proust’s description and studies carried out in animals with the use of retrieval cues. This analysis leads to formulating the hypothesis of the existence of complex interactions between emotion and involuntary retrieval for the autobiographical memory that remains to be further explored by neuroscientists in humans and in animals.

## The madeleine episode

“*I lifted to my lips a spoonful of the tea in which I had left a piece of madeleine to soften.”* For the lay person, this action, which takes place at the beginning of the first volume of *In Search of Lost Time*, is often the only extract they retain from the immense work, seven volumes, more than two thousand pages, by Proust (1871–1922). In this passage, the writer, who complains of his lack of memory, reports on a taste experience that allowed him to rediscover memories of his childhood that he thought were lost forever. After ingesting a piece of the soft cupcake, the author painstakingly describes his reactions. Everyone finds there, often without having read the text, the illustration of a phenomenon commonly experienced, which allows us consciously, through information, a cue, to find a forgotten memory. But, as we will see, this is not what Marcel Proust describes in this passage. When he tastes this madeleine, he has no desire to act on his memory. The cue presents itself to him outside of any conscious search strategy: “*The very moment the sip mixed with the crumbs of the cake touched my palate, I started, attentive to what was extraordinary happening in me*.” Proust is seized by sensory information that triggers an attentional awakening: *“A delicious pleasure had invaded me, isolated, without the notion of its cause.”* This attentional awakening leads to the appearance of a pleasant emotion: “*I felt that it was linked to the taste of tea and cake, but that it exceeded it infinitely, should not be of the same nature. Where was it from? “*. Gradually, the narrator understands that the emotion is linked to the taste of the madeleine, but he finds no connection between the cue and the emotion, and notes that the effect of the cue diminishes with repetition. He realizes that it is futile to try to reactivate the memory with his consciousness. *“leave that and drink my tea simply thinking of my troubles today, my desires for tomorrow.”* You have to let the mind work and trust it: *“And suddenly the memory appeared. This taste was that of the little piece of madeleine that on Sunday mornings at Combray (because that day I did not go out before mass time), when I went to say hello to her in his room, my Aunt Léonie offered it to me after having dipped it in her infusion of tea or lime blossom. ».*

As the narrator has abandoned conscious search processes, memory emerges. This particular mode of retrieval, which Marcel Proust called “involuntary reminiscence,” corresponds to an involuntary retrieval of autobiographical memories, via a sensory cue providing an emotional arousal. What is surprising is that this special process of involuntary memory and emotion, depicted in 1913, for many years received relatively limited research from memory specialists, emphazing the need to pay homage to the visionary nature of Proust.

### Proust: a pioneer whose work anticipated modern neuroscience

Marcel Proust is not a researcher in neuroscience, a discipline whose birth dates back to the end of the 1960s, but, to build his work, he had a vital need to understand how his memory works in order to use it best. To be convinced of this, let us return to the conditions in which this work was written. Marcel Proust came from a bourgeois family. His mother, Jeanne Weil, came from a wealthy family and his father, Adrien Proust, was a renowned professor of medicine. The family fortune ensured the young Marcel an easy existence which, after solid literary studies, and despite his health problems, allowed him to frequent the salons of the upper middle class and the aristocracy of Faubourg Saint Germain and Faubourg Saint Honoré. This social life is punctuated by numerous outings and a few trips.

It was only at the age of 38 that he began writing *In Search of Lost Time*. Proust is asthmatic and his health is fragile. His state of health worsened after the death of his mother (1905). He sets up his own rules of hygiene of life: he gets up at 11 o’clock in the evening to go to bed only at two o’clock the next day because he suffers less at night from his asthma. To manage to fall asleep, he takes sleeping pills in very large quantities, in particular veronal. He consumes a lot of caffeine. For 13 years, that is to say until his death, the man of letters lived as a recluse in his room lined with cork, on the second floor of 102 boulevard Haussmann, in Paris. Alone, locked in his room and bedridden, he gave shape to his literary work. He is exhausted at work, sleeps during the day and rarely goes out, and then always after dark. During all these years of intense writing, he draws on his memory to find the material for his writings. He transposes the anecdotes, events and emotions experienced by himself and those around him during his childhood and during his life as a Parisian dandy to the more than 200 characters populating his “*Research*.” He then realizes that the excessive use of medication, in particular the excessive use of psychotropic drugs such as benzodiazepines, alters his memory and his retrieval abilities. Because the reactivation of his memories is essential to nourish his work, he became interested in memory and more particularly in one of its key stages: the retrieval of memories. During this apprenticeship, Proust observes that the bringing into play of voluntary memory driven by conscious research processes is disappointing as “*it only gives rise to a restricted and imperfect restitution of the initial event*,” as he confided in an interview with the journalist Élie Joseph Bois, in 1913 (see Dyer, 2013). For Proust, voluntary memory is “*a memory of the intelligence and the eyes, which only gives us faces of the past without truth*.” This conscious retrieval only gives access to a past “*different from what we thought we remembered*.” It was not until 1932 that Frederic C. Bartlett, a British precursor of cognitive psychology, joined this observation. Based on experiments, Bartlett proposed that recall is a reconstruction^1^ that leads to an approximate and sometimes erroneous form of the initial memory. This is due to distortions induced by the influence of cultural knowledge and by the intrusion of elements related to the conditions of recall. To voluntary memory, Marcel Proust contrasts involuntary memory, which is implemented by “*a smell, a flavor found in completely different circumstances, [.] despite ourselves*.” The writer finds that allowing this involuntary memory to come into play, as described in the episode of the madeleine, is particularly effective. It is about paying attention to the small sensory information that surround us. When these cues capture our attention and induce an emotion, it is advisable to let your mind find, by itself, the memory which is attached to it. He uses this “protocol” on several occasions in the rest of his work and especially in the last volume, *Finding Time Again* (published posthumously in 1927).

In his interview with Élie Joseph Bois, Marcel Proust repeatedly emphasized that what he considered essential in his work was “*the distinction between voluntary and involuntary memory*.” This formula does not refer to the formation of memory, but to the retrieval of memories. He proposes to consider a form of retrieval brought into play by a sensory cue encountered by chance, which makes it possible to recover an autobiographical memory without engaging conscious search processes, i.e., an involuntary conscious memory. As we have noted, Proust speaks of “*involuntary reminiscence*,” that is to say, of a spontaneous reactivation of a memory. Such an insight in 1913 was very innovative, considering the state of the experimental study of human memory.

Admittedly, Ebbinghaus (1885) distinguished three basic kinds of memory (cited by Tulving, 1985): *voluntary conscious memory*, *involuntary conscious memory* and *involuntary unconscious memory*. Nevertheless, there is little chance that Proust consulted this work, and indeed for almost a century, researchers in the field have considered only two forms of memory: the *voluntary conscious* and the *involuntary unconscious memory.*

Alongside the conscious versus unconscious aspect, Proust was among the first to point out that exposure to cues can induce autobiographical memory recall. He pointed out that the smell was certainly more specific than the sight which was encountered more often: “*The sight of the little madeleine had reminded me of nothing before I had tasted it; perhaps because, having often seen since then, without eating it, on the shelves of pastry chefs, their image had left those days of Combray to link to other more recent ones …*”. Once again, Proust presciently underlined that the specificity of the retrieval cue is a key factor for its effectiveness as a reminder.

Since the publication of *In Search of Lost Time*, numerous studies have been carried out on the evocative power of sensory cues. It can be noted, however, that these began nearly eighty years later. One of the pioneers in this field, the Canadian psychologist Rachel Herz, published an article in 1992 whose aim was to study the powerful significance of odors on the recall of memories (Herz and Cupchik, 1992). The many other articles that followed, at the crossroads of perception, emotion and cognition, have shown the strong evocative and emotional power of odors, which generally exceeds that of other sensory cues such as visual or auditory information (Chu and Downes, 2000; Larsson and Willander, 2009; de Bruijn and Bender, 2018; for a review see Saive et al., 2014).

However, although some of these latter studies evoke the episode of Proust’s madeleine, the subjects must, in most cases, engage explicit/conscious research processes using cues provided by the experimenters and therefore do not reproduce the conditions of the madeleine episode.

It was not until 1985 (more than seventy years after Proust) that two cognitive scientists, Graf and Schacter (1985), proposed a dichotomy of memory between explicit (conscious) and implicit (unconscious/automatic) forms of memory, respectively supported by intentional and unintentional retrieval. The first instance of implicit memory is often attributed to Claparède (1907); see Nicolas (1996) whose amnesic patient withdrew her hand when he met her again. As it turned out, he had previously pricked her with a pin hidden in his hand, altough she had no conscious memory of this episode. For Ebbinghaus and later Graf and Schacter, the *involuntary unconscious* form of recall described in humans, is very different from the Proust’s “*involuntary reminiscence*.” Implicit memory, as seen in the example from Claparède, refers to a general and unconcious phenomenon whereby exposure to one stimulus may influence a response to a subsequent stimulus, without any reference to the initial episode. Under laboratory conditions, implicit memory is often studied with “priming” tests. For instance, when the first three letters of a word previously presented in a list of words is delivered to a subject who is asked to give the first word that comes to mind, without reference to the list presented beforehand, the subject frequently gives the word from the previous list, whereas the primer also corresponds to several words which were not in the list. Implicit memory depends on the existence of some relationship between two stimuli and does not apply for autobiographical memory, because it is not related to any previous memory.

Concerning voluntary conscious memory, although different types of tasks have been used, many experiments have involved presentation of word lists or other material to subjects who were instructed to study them (Radvansky, 2006; Schwartz, 2020). Three types of memory tests engage consciousness. In a free recall test, subjects are asked to give words they remenber; in cued recall tests, semantic cues are delivered to the subject (animal, furniture, color) just before each word recall, and in a recognition test, a word from the list is presented together with another word and the subject must recognize which word was in the initial list. Typically, free recall is more difficult than cued recall, and that cued recall is more difficult than recognition. In all these cases, conscious retrieval processes are engaged. In the minds of the lay person, and in most of the scientific analyses, the episode of the madeleine is often assimilated to a form of cued recall, the taste information driving the active search for the memory (Chu and Downes, 2000). However, as previously emphasized, Proust noted that during the madeleine episode, these active and conscious processes were ineffective. In fact, for many years, researches overlooked the *involuntary conscious memory* corresponding to what Proust described. It is worth mentioning that Semon (1904); see Schacter et al. (1978) developed the concept of ecphory (later adopted by Tulving) to describe a memory retrieval induced by a cue that matches information stored in memory, and enables the conscious access to that memory. However, as with Ebbinghaus’s, there is little chance that Proust was aware of these works. Schacter (1998) noted that Proust’s *“involuntary recalls”* are a common experience that requires *“no deliberate attempt at effort to think back to the past,”* without, however, making it a particular category.

It was not until the 2000s that cognitive psychologists began to consider the third category of memory evoked by Ebbinghaus, the involuntary conscious memory. Berntsen (1996, 2007) introduced the concept of involuntary autobiographical memories (IAM) that occurs when cues encountered in everyday life evoke recall of past events which spring to mind quickly and uncontrollably, without conscious effort (Mace, 2014). Various cues can trigger IAM. In a carefully conducted study, Mace (2004) investigated the types of cues that led to involuntary autobiographical memories. The initiating stimuli were classified as internal or external, which in turn were combined with abstract, sensory/perceptual, or state categories. The major finding, contrasting with Proust’s position, was that only about 30% of the IAMs were classified as sensory in nature, and fewer of the particular cues (odor, taste) that Proust described. However, sensory cues tend to elicit more emotional memories and evoke stronger feelings than other types of cues (see Berntsen, 2007, 2021). As proposed by Proust, Berntsen (1996, 2010) underlined that involuntary autobiographic memories were more specific, less frequently rehearsed, more vivid and more emotionally positive than the voluntary memories. Retrieval time for such involuntary memories is shorter than for strategically retrieved events. The concept of voluntary/involuntary memory is similar to the one of direct versus generative retrieval proposed by Conway (2005).

Up to now, it has not been reported that these various cues induce any emotion preceding the recall of the associated event, except in certain conditions. Intrusive memory, considered as signs of symptoms associated with stress pathologies such as post-traumatic stress disorder (Horowitz, 1976; Bernsten, 2021), are now considered as IAMs, since they are generally triggered unconsciouly by cues associated with the traumatic episode. Interestingly, these cues induce strong emotions (threat and anxiety) as well as vivid flashbacks of the traumatic event. Cues associated with drug taking are also well known to induce involuntary and uncouscious consequences such as craving (Shaham et al., 2003; Vafaie and Kober, 2022).

Exposure to cues has been found to induce partial recovery from organic amnesia in two patients with extremely dense retrograde amnesia (Lucchelli et al., 1995), supporting the notion that IAM result from unconscious processes.

For a long time, involuntary autobiographical memories were only considered by artists, such as Proust (see Tadié, 1998). They began to receive systematic attention from cognitive scientists about 25 years ago, well after Proust’s insightful observations (see Berntsen, 1996, 2021; Mace, 2014; Barzykowski and Moulin, 2022; Moulin et al., 2023).

### The contribution of data obtained in animals

In animals, retrieval is inferred from returning the subjects to the learning situation and measuring performance. In animal research, retrieval of information is used in part to evaluate the level of retention after short or long term retention intervals. A more central reason is to analyse the effects of various treatments on the formation of memory. This approach is based on the fact that, since its early beginnings, animal reseach has largely focussed on the encoding phase and storage processes. As a consequence, different types of retrieval processes have not typically been considered. Nevertheless, it has been well demonstrated that memories involved in different types of tasks are supported by different cerebral circuits, including the hippocampus, a brain structure known to be critically involved in humans in conscious memory. Such a finding led several researchers to distinguish explicit-like and implicit-like tasks (e.g., Eichenbaum, 1997), in order to suggest a similarity between animal and human findings. However, this position is still controversial for many cognitive psychologists, who traditionally have considered that explicit and/or autobiographical memory applies only in humans (e.g., Tulving, 1983; Davachi and Dobbins, 2008).

#### Retrieval cues

Memory recovery subsequent to exposure to partial retrieval cues (also termed reminders^2^), a situation close to the one depicted by Proust, has been explored in several circumstances in animals. For instances, Martin-Ordas et al. (2013) showed that chimpanzees and orangutans, having experienced several years earlier a reinforced situation involving hidden tools, were able, when presented with a specific cue associated with the initial event, to remember the precise location of the tool. This study may suggest that, as in Proust’s example, exposure to retrieval cues in apes is able to favor the retrieval of an associated memory, mirrorring some of the features of human involuntary autobiographical memories (for similar results, see also Lewis et al., 2017).

Numerous kinds of evidence consistent with that finding has been provided in rodent research. A myriad of experiments have shown that exposure to certain cues shortly before a retention test facilitated the retrieval of information whose access had been disrupted by amnesic treatments delivered just after learning (see Miller and Springer, 1972; Spear, 1973; Miller et al., 1986; Gisquet-Verrier and Riccio, 2018, 2019, 2022 for more details). Exposure to these cues also reduced spontaneous performance impairments, such as long-term forgetting or infantile amnesia and even improved animal performance at testing in the absence of any disruption (e.g., Campbell and Jaynes, 1966; Spear, 1973; Moye and Thomas, 1982; Gisquet-Verrier and Riccio, 2012 for more details). Although most of these data were obtained in rodents, similar results have been described in many other animal species (vertebrates and invertebrates). Taken together, these data clearly show that exposure to retrieval cues are able to promote retrieval and enhance access to memories when either spontaneously or experimentally disrupted.

As with humans, various types of information specific to the initial training situation such as sensory cues, contextual cues or even internal cues (induced by hunger, thirst or various drugs) have been used as reminders, i.e., providing effective cues to promote retrieval processes. The few studies that have compared the effectiveness of these various cues over time show that it varies according to the length of retention of information. The characteristics of the cues must be precise and very close to the initial information when delivered soon after learning, while more global ones, such as context, can be effective long after training (Gisquet-Verrier et al., 1989; Mac Ardy and Riccio, 1991; Spear and Riccio, 1994). Interestingly, as in humans, exposure to trauma associated cues induces threat and anxiety, while exposure to drug associated cues induces drug craving (see Gisquet-Verrier, 2009; Le Dorze and Gisquet-Verrier, 2016).

#### Reminders and emotion

Studying the time course of facilitative effects induced by the retrieval cues, it was observed that their effects were not obtained immediately but required several minutes to be effective (Gisquet-Verrier and Alexinsky, 1990). Because damage to the amygdala, a key structure in emotional processes, blocks the effectiveness of retrieval cues (Baker et al., 1981), we suspected that emotion was potentialy involved in this situation. The analyses of blood samples taken from animals after five minutes following exposure to a reminder indicated increases in ACTH and corticosterone levels only when the cues were effective in generating retrieval, reflecting an increase in the animals’ emotionality (Gisquet-Verrier et al., 2004), and accounting for the delay after which cues are effective.

To explain the link between emotion and memory retrieval, it has been proposed that the presentation of a retrieval cue induces a kind of truncated conditioned reflex that results in a release of norepinephrine (Sara and Bouret, 2012), which, by increasing attention, initiates a hormonal cascade leading to the facilitation of memory retrieval.

#### Cerebral circuit supporting reminders effects

Cerebral structures involved during cue-elicited retrieval processes have also been investigated using various approaches, including lesions and measures of metabolic activities. This research confirmed the role of the amygdala and further indicated a role of several other brain structures, including some structures known to release neurotransmitters (raphe nucleus, ventral tegmental area, hypothalamus; Boujabit et al., 2003), as well as brain structures known to be involved in the achievement of retrieval processes (prelimbic and anterior cingulate cortex; Botreau et al., 2004). As in humans, reminder cues are still effective in rats with lesions to the hippocampus (Gisquet-Verrier and Schenk, 1994), suggesting that reminders largely rely on involuntary process in animals.

## Conclusion

Because Proust did not have any psychological background, and worked primarily as a writer, one might be surprised at his relevance, cut off from the world and experimenting only on himself (interestingly, Ebbinghaus also was often the only subjects of his sudies).

This view does not take into account the influence of his time, of his scholarly milieu and of his family. Proust’s father was at the time a renowned doctor, at the forefront of the latest scientific and medical discoveries. Before his isolation, Proust also frequented Parisian salons which allowed him to rub shoulders with enlightened and varied personalities. Proust is a contemporary of the philosopher Henri Bergson (1859–1941), with whom he was close and who worked on memory (Bergson, 1896). And although their conceptions on this subject do not overlap, as noted in the interview with the journalist Élie Joseph Bois (see Dyer, 2013), Bergson exerted a great influence on the writer. Proust is also the contemporary of Sigmund Freud (1856–1939) and, if they never met, we can suggest that involuntary memory was probably largely inspired by the notion of the unconscious developed by Freud at the same period of time.

In the last volume of “*The Research*,” Proust experiments with the power of involuntary reminiscences by eliciting the resurgence of the past from various kinds of sensory information encountered during his daily life. In “*Time Regained*,” the writer thus multiplies the examples of memories obtained thanks to involuntary memory, recounting a series of happy moments: the stumble caused by a street pavement slightly askew reminds him of the baptistery of Saint Mark and the pleasure he had in Venice; the smack of a spoon against a plate evokes the sound of a hammer on a wheel of a railway car he heard while he was with Albertine; the touch of a starched napkin brings back his arrival at Balbec (a seaside resort in Normandy, where the narrator had happy holidays), the sound of driving in water merges with the noise of pleasure boats he liked to observe. For all of these episodes Proust reports the emotion that precedes the recall and the happiness felt at being able to bring back the living memory of a past episode. Thus, by intensifying the attention paid to the small cues that life spontaneously offers him, Proust was able to reactivate and enjoy many old memories. His approach shows that any type of sensory information can be used to promote this resurgence, whether it be auditory (the smack of the spoon), kinesthetic (the pavement), somesthetic (the stiffness of the napkin) or visual (such as the view of the steeples of Martinville, in another volume of his work).

This article describes Proust’s numerous intuitions (later validated) concerning memory processes in general, supporting the idea proposed by Jonah Lehrer that Proust should be considered the first “neuroscientist” (Lehrer, 2007).

For the record, Proust proposed that memory is a reconstruction. He emphasized that retrieval cues can induce a recall of memory and that any sensory cues may serve as reminders, with olfactory cues being more effective than less specific visual cues, for remote memory. He suggested that autobiographic memory can be accessed either by voluntary or involuntary processes and that memories recalled by involuntary process are more vivid than those obtained with voluntary process.

As underlined in several occasions in the present paper, Proust demonstrated clear visionary intuitions concerning the functioning of memory. It has not been widely recognized that he is important to the origin of the concept of involuntary memory, i.e., allowing memories to be revived without having to reconstruct them using voluntary memory. Despite the fact that it remains difficult to assimilate the Proust’s madeleine episode to memory recovery in animals exposed to retrieval cues, we can, however, emphasize a number of convergences. In both situations, exposure to some cues is able to promote processes which favor the retrieval of an associated memory. These cues can be various sensory cues, or even internal cues. In both situations, an emotional reaction seems to be required. In both cases, the effects are not immediate but require some time to be effective in generating the retrieval. Finally, in both cases, the effects of the cues do not seem to rely on the integrity of the hippocampus, a structure known to play a determinant role in voluntary retrieval.

Although Proust’s memory recovery was very much like what are now called IAMs (for a review see Berntsen, 1996, 2021; Mace, 2014; Barzykowski and Moulin, 2022), the Madeleine episode had two somewhat different features: 1. the return of memory is not immediate but requires a period of time and 2. there is a strong emotional component involved in the recall of memory. Interestingly, these two features have been also demonstrated in animals’ studies. For example, it has been shown that direct retrieval induced by emotional cues takes longer than with object cues (Uzer et al., 2012). However, the idea suggested by Proust that an emotion elicited by a retrieval cue may trigger an involuntary retrieval process, has still not been studied in humans, and only considered in a few studies on animals.

That mood may direct retrieval of congruent memories is well documented in human (Blaney, 1986), as well as in animals (Harding et al., 2004). The effects of reminder cues may well illustrate the encoding specificity principle (Tulving and Thomson, 1973), which postulates that correct recall occurs when some stimuli present during retrieval correspond with some features of the initial events. This principle is also illustrated in drug state dependent research on memory, where the internal state at testing needs to match the state present at training for successful retrieval of information (Overton, 1991).

The Madeleine episode begins by an attempt at a voluntary retrieval which did not succeed, followed by an involuntary search process which led to the memory recall. Recently, Moulin et al. (2023) proposed that autobiographical memories must exceed a threshold to be retrieved and this might be achieved in mixing voluntary and involuntary processes in an iterative fashion. Such a process may take some time to lead to retrieval and suggest that memories should not be classified as voluntary or involuntary but exist on the same continuum.

What we propose here is that relevant sensory information, when added to emotion, exert a cumulative effect toward reaching the retrieval threshold, enabling direct access to otherwise less retrievable memories through involuntarily processes. Such an effect, which does not involve consciousness, can be considered as a basic phenomenon for retrieval, constituting a distinct category of involuntary retrieval for autobiographical memories. Such a view has two main consequences: first, to introduce a new form of IAM retrieval in humans; and second, to consider that animals, as humans, may have autobiographical memories (e.g., Millin and Riccio, 2019).

After nearly one century following Proust‘s death, cognitive psychologists now recognize the reality of involuntary autobiographic memories. Recent research development on IAMs indicates that these memories are much more frequent than initially thought and constitute a basic mode of remembering past events. Thus, it might be an appropriate time for neuroscientists to increase their interest in this new form of recall and elucidate its neural basis, as this type of memory seems universal enough to be considered not only in humans but also in animals.

## Author contributions

PG-V had the idea and wrote a first draft. DR completed and provided his advice and knowledge. All authors contributed to the article and approved the submitted version.

## Footnotes

^1^Bartlett (1932, pg. 213) pointed out that retrieval does not correspond to the reactivation of fixed traces but correspond to a reconstruction at the time of retrieval, accounting for biases and inconsistencies that may appear during the recollection of memories.

^2^In animal studies, the term “reminders” typically refers to a cue (e.g., conditioned stimulus) used in training that is not followed by the original outcome (unconditioned stimulus). As Sinclair and Barense (2019) have noted in examining the important role of error prediction in memory, this arrangement would be considered an incomplete reminder in human studies.

## References

[ref1] Baker L. J. Kesner R. P. Michal R. E. (1981). Differential effects of a reminder cue on amnesia induced by stimulation of amygdala and hippocampus. J. Comp. Physiol. Psychol. 95, 312–321. doi: 10.1037/h0077773 7229162

[ref2] Bartlett F. C. (1932). Remembering: a study in experimentaland social psychology. London: Cam.

[ref3] Barzykowski K. Moulin C. (2022). Are involuntary autobiographical memory and déjà vu natural products of memory retrieval? Behav. Brain Sci. 46:e356. doi: 10.1017/S0140525X22002035, PMID: 36111499

[ref4] Bergson H. (1896). Matière et mémoire [matter and memory]. Paris: Alcan.

[ref5] Berntsen D. (1996). Involuntary autobiographical memories. Appl. Cogn. Psychol. 10, 435–454. doi: 10.1002/(SICI)1099-0720(199610)10:5<435::AID-ACP408>3.0.CO;2-L

[ref6] Berntsen D. (2007). “Involuntaryautobiographical memories: speculations, findings and an attempt to integrate them” in Involuntary memory. ed. Mace K. J. (Malden, MA: Blackwell), 20–50.

[ref7] Berntsen D. (2010). The unbidden past: involuntary autobiographical memories as a basic mode of remembering. Curr. Dir. Psychol. Sci. 19, 138–142. doi: 10.1177/0963721410370301

[ref8] Berntsen D. (2021). Involuntary autobiographical memories and their relation to other forms of spontaneous thoughts. Philosophical transactions of the Royal Society of London. Ser. B Biol Sci 376:20190693. doi: 10.1098/rstb.2019.0693, PMID: 33308074 PMC7741080

[ref9] Blaney P. H. (1986). Affect and memory: a review. Psychol. Bull. 99, 229–246. doi: 10.1037/0033-2909.99.2.229 3515383

[ref10] Botreau F. El Massioui N. Chéruel F. Gisquet-Verrier P. (2004). Effects of medial prefrontal cortex and dorsal striatum lesions on retrieval processes in rats. Neuroscience 129, 539–553. doi: 10.1016/j.neuroscience.2004.08.032 15541876

[ref11] Boujabit M. Bontempi B. Destrade C. Gisquet-Verrier P. (2003). Exposure to a retrieval cue in rats induces changes in regional brain glucose metabolism in the amygdala and other related brain structures. Neurobiol. Learn. Mem. 79, 57–71. doi: 10.1016/s1074-7427(02)00010-2, PMID: 12482680

[ref12] Campbell B. A. Jaynes J. (1966). Reinstatement. Psychol. Rev. 73, 478–480. doi: 10.1037/h0023679 5976739

[ref13] Chu S. Downes J. J. (2000). Odour-evoked autobiographical memories: psychological investigations of proustian phenomena. Chem. Senses 25, 111–116. doi: 10.1093/chemse/25.1.111, PMID: 10668001

[ref14] Conway M. A. (2005). Memory and the self. J. Mem. Lang. 53, 594–628. doi: 10.1016/j.jml.2005.08.005

[ref15] Davachi L. Dobbins I. G. (2008). Declarative memory. Curr. Dir. Psychol. Sci. 17, 112–118. doi: 10.1111/j.1467-8721.2008.00559.x, PMID: 20011622 PMC2790294

[ref16] de Bruijn M. J. Bender M. (2018). Olfactory cues are more effective than visual cues in experimentally triggering autobiographical memories. Memory 26, 547–558. doi: 10.1080/09658211.2017.1381744, PMID: 28969504

[ref18] Dyer N. M. (2013). Le lancement de" Du côté de chez Swann": Brouillon de l'«entretien» de novembre 1913 avec Élie-Joseph Bois. Bulletin d'informations proustiennes 43, 9–22.

[ref19] Ebbinghaus H. (1885). Über das Gedächtnis: Untersuchungen zur experimentellen Psychologie. Duncker & Humblot, Leipzig 1885.

[ref20] Eichenbaum H. (1997). Declarative memory: insights from cognitive neurobiology. Annu. Rev. Psychol. 48, 547–572. doi: 10.1146/annurev.psych.48.1.547, PMID: 9046568

[ref21] Gisquet-Verrier P. (2009). “Hypersensitivity to cue-elicited memory reactivation as a possible source for psychiatric pathologies such as relapse to drug addiction and post traumatic stress disorder” in Endophenotype and psychiatric and neurodegenerative disorders in animal models. ed. Granon S. (Trivandrum, Kerala, India: Transworld Research Network), 41–82.

[ref22] Gisquet-Verrier P. Alexinsky T. (1990). Long-term spontaneous improvement of performance is related to the strength of the initial training: theoretical implications. Behav. Neural Biol. 53, 298–304. doi: 10.1016/0163-1047(90)90577-S 2331238

[ref9001] Gisquet-Verrier P. Riccio D. C. (2012). Memory reactivation effects independent of reconsolidation. Learning and memory (Cold Spring Harbor, N.Y.), 19, 401–409. doi: 10.1101/lm.026054.112 22904371

[ref23] Gisquet-Verrier P. Riccio D. C. (2019). Memory integration as a challenge to the consolidation/reconsolidation hypothesis: similarities, differences and perspectives. Front. Syst. Neurosci. 12:71. doi: 10.3389/fnsys.2018.00071 30687031 PMC6337075

[ref24] Gisquet-Verrier P. Riccio D. C. (2018). Memory integration: an alternative to the consolidation/reconsolidation hypothesis. Prog. Neurobiol. 171, 15–31. doi: 10.1016/j.pneurobio.2018.10.002, PMID: 30343034

[ref25] Gisquet-Verrier P. Riccio D. C. (2022). Revisiting systems consolidation and the concept of consolidation. Neurosci. Biobehav. Rev. 132, 420–432. doi: 10.1016/j.neubiorev.2021.12.003, PMID: 34875279

[ref26] Gisquet-Verrier P. Schenk F. (1994). Selective hippocampal lesions in rats do not affect retrieval processes promoted by prior cuing with the conditioned stimulus or the context. Psychobiology 22, 289–303. doi: 10.3758/BF03327112

[ref27] Gisquet-Verrier P. Botreau F. Venero C. Sandi C. (2004). Exposure to retrieval cues improves retention performance and induces changes in ACTH and corticosterone release. Psychoneuroendocrinology 29, 529–556. doi: 10.1016/s0306-4530(03)00085-4, PMID: 14749097

[ref28] Gisquet-Verrier P. Dekeyne A. Alexinsky T. (1989). Differential effects of several retrieval cues over time: evidence for time-dependent reorganization of memory. Anim. Learn. Behav. 17, 394–408. doi: 10.3758/BF03205220

[ref29] Graf P. Schacter D. L. (1985). Implicit and explicit memory for new associations in normal and amnesic subjects. J. Exp. Psychol. Learn. Mem. Cogn. 11, 501–518. doi: 10.1037//0278-7393.11.3.501 3160813

[ref30] Harding E. J. Paul E. S. Mendl M. (2004). Cognitive bias and affective state. Nature 427:312. doi: 10.1038/427312a 14737158

[ref31] Herz R. S. Cupchik G. C. (1992). An experimental characterization of odor-evoked memories in humans. Chem. Senses 17, 519–528. 10.1093/chemse/20.5.517 8564426

[ref32] Horowitz MJ. (1976). Stress response syndromes. Oxford, UK: Jason Aronson.

[ref33] Larsson M. Willander J. (2009). Autobiographical odor memory. Ann. Acad. Sci 1170, 318–323. doi: 10.1111/j.1749-6632.2009.03934.x 19686154

[ref34] Le Dorze C. Gisquet-Verrier P. (2016). Effects of multiple brief exposures to trauma-associated cues on traumatized resilient and vulnerable rats. Brain Res. 1652, 71–80. doi: 10.1016/j.brainres.2016.10.002, PMID: 27717871

[ref35] Lehrer J. (2007) Proust was a neuroscientist. Houghton Mifflin Harcourt (Eds). New York.

[ref36] Lewis A. Call J. Bernsten D. (2017). Non goal-directed recall of specific events in apes after long delays. Proc. R. Soc. B 27, 38–48. doi: 10.1080/09658211.2017.1363243 PMC5524493 28701556

[ref37] Lucchelli F. Muggia S. Spinnler H. (1995). The ‘petites madeleines’ phenomenon in two amnesic patients: sudden recovery of forgotten memories. Brain 118, 167–181. doi: 10.1093/brain/118.1.167, PMID: 7895003

[ref38] Mac Ardy E. A. Riccio D. C. (1991). Increased generalization between drug-related interoceptive stimuli with delayed testing. Behav. Neural Biol. 56, 213–219. doi: 10.1016/0163-1047(91)90608-s, PMID: 1759942

[ref39] Mace J. H. (2004). Involuntary autobiographical memories are highly dependent on abstract cuing: the Proustian view is incorrect. Appl. Cogn. Psychol. 18, 893–899. doi: 10.1002/acp.1020

[ref40] Mace J. H. (2014). Involuntary autobiographical memory chains: implications for autobiographical memory organization. Front. Psych. 5:183. doi: 10.3389/fpsyt.2014.00183 PMC4267106 25566102

[ref41] Martin-Ordas G. Berntsen D. Call J. (2013). Memory for distant past events in chimpanzees and orangutans. Curr. Biol. 23, 1438–1441. doi: 10.1016/j.cub.2013.06.017, PMID: 23871242

[ref42] Miller R. R. Springer A. D. (1972). Induced recovery of memory in rats following electroconvulsive shock. Physiol. Behav. 8, 645–651. doi: 10.1016/0031-9384(72)90089-3, PMID: 5064524

[ref43] Miller R. R. Kasprow W. J. Schachtman T. R. (1986). Retrieval variability: sources and consequences. Am. J. Psychol. 99, 145–218. doi: 10.2307/1422275 2876652

[ref44] Millin P. M. Riccio D. C. (2019). False memory in nonhuman animals. Learn. Mem. 26, 381–386. doi: 10.1101/lm.050054.119, PMID: 31527184 PMC6749930

[ref45] Moulin C. J. A. Carreras F. Barzykowski K. (2023). The phenomenology of autobiographical retrieval, Wiley interdisciplinary reviews. Cogn. Sci. 14:e1638. doi: 10.1002/wcs.1638, PMID: 36458642

[ref46] Moye T. B. Thomas D. R. (1982). Effects of memory reactivation treatments on postdiscrimination generalization performance in pigeons. Anim. Learn. Behav. 10, 159–166. doi: 10.3758/BF03212264

[ref47] Nicolas S. (1996). Experiments on implicit memory in a Korsakoff patient by Claparede (1907). Cogn. Neuropsychol. 13, 1193–1199. doi: 10.1080/026432996381700

[ref48] Radvansky G. (2006). Human memory. Boston, Massachusetts: Pearson Publisher.

[ref49] Overton D. A. (1991). Historical context of state dependent learning and discriminative drug effects. Behav. Pharmacol. 2, 235–264. doi: 10.1097/00008877-199109000-00002 11224069

[ref50] Saive A. L. Royet J. P. Plailly J. (2014). A review on the neural bases of episodic odor memory: from laboratory-based to autobiographical approaches. Front. Behav. Neurosci. 8:240. doi: 10.3389/fnbeh.2014.00240, PMID: 25071494 PMC4083449

[ref51] Sara S. J. Bouret S. (2012). Orienting and reorienting: the locus coeruleus mediates cognition through arousal. Neuron 76, 130–141. doi: 10.1016/j.neuron.2012.09.011 23040811

[ref52] Schacter D. L. (1998). Memory and awareness. Science 280, 59–60. doi: 10.1126/science.280.5360.59 9556454

[ref53] Schacter D. L. Eich J. E. Tulving E. (1978). Richard Semon's theory of memory. J. Verb. Learn. Verb. Behav. 17, 721–743. doi: 10.1016/S0022-5371(78)90443-7

[ref54] Schwartz B. L. (2020). Memory: Foundations and applications. Thousands Oaks, California: Sage Publications.

[ref9002] Semon. (1904). Die Mneme als erhaltendes Prinzip im Wechsel des organischen Geschehens, Wilhelm Engelmann, Leipzig.

[ref55] Shaham Y. Shalev U. Lu L. de Wit H. Stewart J. (2003). The reinstatement model of drug relapse: history, methodology and major findings. Psychopharmacology 168, 3–20. doi: 10.1007/s00213-002-1224-x 12402102

[ref56] Sinclair A. H. Barense M. D. (2019). Prediction error and memory reactivation: how incomplete reminders drive reconsolidation. Trends Neurosci. 42, 727–739. doi: 10.1016/j.tins.2019.08.007, PMID: 31506189

[ref57] Spear N. E. (1973). Retrieval of memory in animals. Psychol. Rev. 80, 163–194. doi: 10.1037/h0034326

[ref58] Spear N. E. Riccio D. C. (1994). Memory: Phenomena and principles. Needham Heights, Massachusetts: Allyn & Bacon.

[ref59] Tadié J.-Y. (1998). Nouvelles recherches sur la mémoire proustienne. Revue des Sciences morales et politiques, Paris, no 4:71.

[ref60] Tulving E. (1983). Elements of episodic memory. New York, NY: Oxford University Press.

[ref61] Tulving E. (1985). Memory and consciousness. Canad. Psychol. 26, 1–12. doi: 10.1037/h0080017

[ref62] Tulving E. Thomson D. M. (1973). Encoding specificity and retrieval processes in episodic memory. Psychol. Rev. 80, 352–373. doi: 10.1037/h0020071

[ref63] Uzer T. Lee P. J. Brown N. R. (2012). On the prevalence of directly retrieved autobiographical memories. J. Exp. Psychol. Learn. Mem. Cogn. 38, 1296–1308. doi: 10.1037/a0028142, PMID: 22545602

[ref64] Vafaie N. Kober H. (2022). Association of Drug Cues and Craving with Drug use and relapse: a systematic review and meta-analysis. JAMA Psychiatry 79, 641–650. doi: 10.1001/jamapsychiatry.1240, PMID: 35648415 PMC9161117

